# Enhancing Image Quality in Dental-Maxillofacial CBCT: The Impact of Iterative Reconstruction and AI on Noise Reduction—A Systematic Review

**DOI:** 10.3390/jcm14124214

**Published:** 2025-06-13

**Authors:** Róża Wajer, Pawel Dabrowski-Tumanski, Adrian Wajer, Natalia Kazimierczak, Zbigniew Serafin, Wojciech Kazimierczak

**Affiliations:** 1 Department of Radiology and Diagnostic Imaging, Collegium Medicum, Nicolaus Copernicus University in Torun, Jagiellońska 13-15, 85-067 Bydgoszcz, Poland; wojtek.kazimierczak@gmail.com; 2 Faculty of Mathematics and Natural Sciences, School of Exact Sciences, Cardinal Stefan Wyszynski University, 01-815 Warsaw, Poland; p.dabrowski-tumanski@uksw.edu.pl; 3 Independent Researcher, 86-005 Zielonka, Poland; adrianwajer1@gmail.com; 4 Kazimierczak Private Medical Practice, Dworcowa 13/u6a, 85-009 Bydgoszcz, Poland; natnowicka@gmail.com; 5 Faculty of Medicine, Bydgoszcz University of Science and Technology, Kaliskiego 7, 85-796 Bydgoszcz, Poland; zbigniew.serafin@pbs.edu.pl

**Keywords:** artifact reduction, cone-beam computed tomography, deep learning model, dental imaging, image quality, iterative reconstruction, noise reduction, oral cavity CBCT imaging

## Abstract

**Background:** This systematic review evaluates articles investigating the use of iterative reconstruction (IR) algorithms and artificial intelligence (AI)-based noise reduction techniques to improve the quality of oral CBCT images. **Materials and Methods:** A detailed search was performed across PubMed, Scopus, Web of Science, ScienceDirect, and Embase databases. The inclusion criteria were prospective or retrospective studies with IR and AI for CBCT images, studies in which the image quality was statistically assessed, studies on humans, and studies published in peer-reviewed journals in English. Quality assessment was performed independently by two authors, and the conflicts were resolved by the third expert. For bias assessment, the Quality Assessment of Diagnostic Accuracy Studies (QUADAS)-2 tool was used for bias assessment. **Material:** A total of eleven studies were included, analyzing a range of IR and AI methods designed to reduce noise and artifacts in CBCT images. **Results:** A statistically significant improvement in CBCT image quality parameters was achieved by the algorithms used in each of the articles we reviewed. The most commonly used image quality measures were peak signal-to-noise ratio (PSNR) and contrast-to-noise ratio (CNR). The most significant increase in PSNR was demonstrated by Ylisiurua et al. and Vestergaard et al., who reported an increase in this parameter of more than 30% for both deep learning (DL) techniques used. Another subcategory used to improve the quality of CBCT images is the reconstruction of synthetic computed tomography (sCT) images using AI. The use of sCT allowed an increase in PSNR ranging from 17% to 30%. For the more traditional methods, FBP and iterative reconstructions, there was an improvement in the PSNR parameter but not as high, ranging from 3% to 13%. Among the research papers evaluating the CNR parameter, an improvement of 17% to 29% was achieved. **Conclusions:** The use of AI and IR can significantly improve the quality of oral CBCT images by reducing image noise.

## 1. Introduction

Cone Beam Computed Tomography (CBCT) has become an essential imaging modality in daily dental practice, providing detailed three-dimensional views of craniofacial structures and aiding in diagnostic, treatment planning, or evaluation of endodontic morphology [1,2,3]. However, CBCT images, more than CT [4], are often affected by noise, artifacts, and other quality issues that can compromise diagnostic accuracy and clinical decision-making [5]. The excess noise generated by CBCT is a result of the conical geometry of the X-ray beam and the lack of post-patient collimation. In addition, higher scattering degrades image quality compared to CT [6], while the commonly used low mAs protocols result in significantly degraded CBCT image quality. Therefore, especially in dental CBCT, image reconstruction plays a key role in improving diagnostic image quality by reducing noise and artifacts while preserving spatial resolution. In 1970, Gordon et al. introduced the first iterative reconstruction (IR) technique for CT scans [7]. However, its usefulness in practical applications was limited due to the high computational demands stemming from the requirement of iterative reprojection and back projection steps, aiming at calculation of the fidelity between measured and estimated projections [8].

To overcome the efficiency problem, a simpler analysis technique called Filtered Back Projection (FBP) has been widely used for more than 40 years [9]. In this technique, only one filtering process and one back projection are used, which speeds up the whole reconstruction process. However, the performance of the FBP technique depends on the quality, distribution, and noise level of the projection data, as well as on the choice of filter. In the 2000s, advanced IR algorithms for CT images were successfully developed to remove noise and artifacts, resulting in high-quality images even with lower-dose protocols, according to the ALARP (As Low As Reasonably Practicable) principle [10], and their effectiveness was confirmed by FDA approval in 2009 [11,12,13,14].

**Figure 1:**
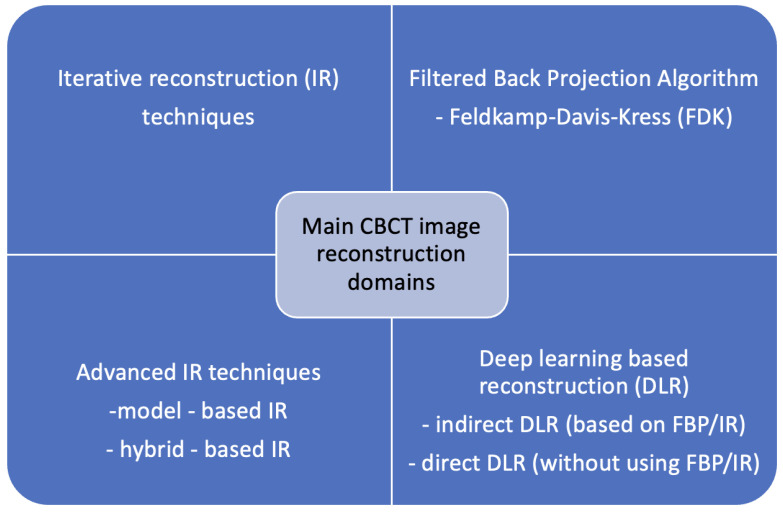
Primary CBCT reconstruction domains.

The adaptation of the FBP technique to cone-beam geometry has mainly been done using the conventional Feldkamp-Davis-Kress (FDK) algorithm, which is prone to image distortions and does not promote the reduction in image noise [15]. It results in blurring or graining the image, which reduces the visibility of critical anatomical features [6,16]. Therefore, an efficient denoising technique is still needed to enhance the difference in the signals between real structures and noise for better CBCT image quality, following their widespread clinical adoption, which is still awaited [17]. Recent advances in artificial intelligence have shown great promise in improving image quality through several deep learning-based reconstruction methods, but the main work is in CT research [18,19,20,21]. Compared to CT studies, the literature on intelligent image denoising methods in CBCT is scarce, but several studies have investigated the use of AI techniques for image quality assessment and enhancement in CBCT. Yet, no systematic review describing those methods was published. Moreover, the suitability of the proposed methods from the AI perspective was never analyzed. The primary CBCT image reconstruction methodologies are presented in Figure 1.

The aim of this systematic review was to critically evaluate the current literature on the role of iterative reconstruction and AI in noise reduction in dental-maxillofacial CBCT. In particular, we attempted to organize and fundamentally group iterative and AI methods to improve noise reduction, image quality improvement, and diagnostic utility in imaging. Moreover, for the first time, we looked at the validity of the methods from an AI perspective and proposed further directions and a framework for evaluating AI performance in CBCT image enhancement.

## 2. Materials and Methods

### 2.1. Article Selection and Data Extraction Process Overview

This systematic review was conducted according to the PRISMA (Preferred Reporting Items for Systematic Reviews and Meta-Analyses) statement [22] and the guidelines of the Cochrane Handbook for Systematic Reviews of Interventions [23]. It was registered within the PROSPERO service with ID 1048103.

**Figure 2:**
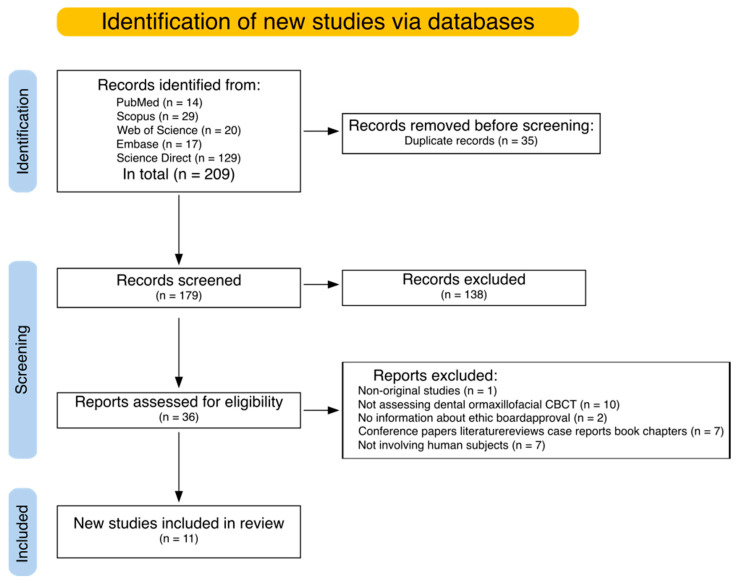
Prisma 2020 flow diagram showing the flow of the studies analyzed, along with the numbers of excluded articles and exclusion reasons.

The initial set of publications, retrieved from search engines, was first deduplicated and then manually screened. The aim of the screen was to (1) exclude articles on different topics that accidentally matched search criteria and (2) select eligible articles based on well-defined criteria, described in a further section. The whole process was shown in Figure 2. To standardize the comparison, each article from the final set was subjected to manual data extraction analysis (described in detail in a further section). Finally, to ensure the objectivity of the results, the Risk of Bias analysis was performed (as described in the further section).

The manual screening, data extraction, and risk of bias analysis were performed independently by two authors (RW and AW). Their agreement was measured by Cohen’s kappa coefficient [24], and the disagreements were resolved by the third expert (WK).

Based on PICO(S) [25], this systematic review concentrated on the following questions: (P) What is the Population (patient groups) in oral and maxillofacial CBCT scans; (I): What was the Intervention, i.e., if the CBCT images were enhanced with or without AI; (C): What was the Control used to assess quantitative and qualitative image enhancement; (O): What was the Outcome after image denoising.

### 2.2. Article Search

A series of preliminary searches of the PubMed, Scopus, Web of Science, ScienceDirect, and Embase databases were performed on 3 January 2025. The final search was performed on 8 January 2025 using all the above search engines. The search phrase was constructed from a combination of MeSH/non-MeSH terms joined by Boolean operators:

*(“CBCT” OR “cone-beam computed tomography”) AND (“denoising” OR “denoise” OR “noise reduction”) AND (“oral cavity” OR “maxillofacial” OR “dental”)*

As the syntaxes of search engines varied, the exact form of the search string was search dependent. The exact form search string for each search engine is present in SI.

### 2.3. Eligibility Criteria

Studies were included if they met the following well-defined Boolean criteria: (1) prospective or retrospective studies with AI for CBCT images, (2) studies in which the image quality was statistically assessed, (3) studies on humans, and (4) studies published in peer-reviewed journals in English. Exclusion criteria were as follows: (a) non-original studies, (b) studies not evaluating diagnostic dental or maxillofacial CBCT, (c) studies evaluating the use of CBCT in radiotherapy and interventional radiology, (d) lack of ethics committee approval, and (e) conference papers, literature reviews, case reports, and book chapters.

### 2.4. Data Extraction

A standardized data extraction form was used to extract data on study characteristics, including author, year, country, sample size, AI method used (deep learning model), dataset partitioning (train/test), quantitative and qualitative evaluation, and anatomical region.

### 2.5. Risk of Bias

The quality of the included studies was assessed using the Quality Assessment of Diagnostic Accuracy Studies (QUADAS)-2 tool [26]. The QUADAS-2 tool encompasses four domains: patient selection, reference standard, flow and timing, and index test. Each domain was assessed for risk of bias, with three domains also assessed for applicability concerns. The assessment of bias is facilitated by signaling questions. The QUADAS-2 methodology was implemented in four sequential steps: firstly, a review question is formulated; secondly, the tool is tailored to provide review-specific guidelines; thirdly, a primary study flowchart is created; and finally, bias and applicability are assessed.

## 3. Results

### 3.1. Search Results

A total of 209 articles were identified as potentially relevant to the subject. Following the removal of 35 duplicates, 174 titles and abstracts were subjected to a rigorous evaluation process. Following this, 138 papers were excluded as they did not meet the inclusion criteria or were not related to the topic of this review. The remaining 36 papers were retrieved and analyzed to perform this review.

The process of searching for relevant literature is illustrated by the PRISMA flowchart (see Figure 1). At this stage, both reviewers demonstrated a high level of agreement, achieving a Cohen’s kappa of 0.98. Discrepancies were resolved by a third reviewer (KW) in a limited number of cases. Thirty-six articles were then screened for full text, of which 25 were excluded for the following reasons: one was a non-original research study, two lacked information on ethical board approval, five were conference papers, literature reviews, case reports, or book chapters; ten studies did not evaluate the assessment of diagnostic dental or maxillofacial CBCT, and seven studies did not involve human subjects. (Supplementary Materials Table S1). Consequently, 11 articles were deemed eligible for inclusion in the review. The data obtained from these studies is presented in Table 1.

The articles and their primary characteristics are summarized in Table 1 and Table 2. Given the heterogeneity in objectives and methodologies across groups, each group is described in a separate section. The majority of studies were conducted in China and Poland (n = 3), followed by Korea and the USA (n = 2), with additional studies from Italy, Denmark, and Finland (n = 1).

### 3.2. Risk of Bias

The risk of bias assessment is summarized in Table 3. In the domain of patient selection, most of the included studies exhibit a low risk of bias, primarily due to the implementation of randomization. The presence of clearly defined inclusion criteria, along with accurate patient data, led to a low risk of bias. Conversely, an absence of complete patient data or clearly defined inclusion criteria resulted in an unclear risk of bias. It has to be noted, however, that although not mentioned explicitly, most probably in all cases the randomization of patients was done retrospectively. The risk was considered high if the study only stated that a certain number of CBCT examinations were included, without specifying their characteristics.

The risk of index test bias was considered low if both intra- and inter-rater agreement were examined. In instances where pertinent information regarding one of these tests was absent, the risk was categorized as unclear. Consequently, in instances where no error study was conducted, the risk was categorized as high.

The risk of bias due to the reference standard was deemed low, as each of the trials had a well-described reference standard, which was usually the FBP projection or the reference CT images. The risk of bias due to flow and timing was considered low in all studies, as the time interval between CBCT and the reference standard was reported. The applicability concerns regarding patient selection remain unaltered due to the nature of the study material.

### 3.3. Study Objectives

The set of retrieved articles is naturally divided into 3 groups, representing 3 different objectives. In the first group, there are two articles seeking to address the denoising problem with iterative refinement or specific filtering algorithms [27,35]. The next group consists of three articles where authors evaluate various aspects of vendor-agnostic, AI-based software for image denoising [28,29,33]. In particular, the authors measure the capability of AI methods to remove metal artifacts or sharpen images in general or in specific regions (temporomandibular joints, root canals). The largest group, consisting of six articles, aims at building its own AI models, aiming at transforming CBCT images to or obtaining them from corresponding CT images or by reconstructing high-quality CBCT images from low-dose samples [15,30,31,32,34,36]. The objectives of each article are summarized in Table 2 and are described in more detail in the following sections.

### 3.4. Metrics and Evaluation

Different objectives require different evaluation metrics. The metrics used in each article are presented in Table 1. In image denoising, one may be interested in how the key features of the original image were preserved. To quantify the similarity between the original and denoised image, most commonly one uses Peak Signal to Noise Ratio (PSNR, most common), Feature Similarity (FSIM), Structure Similarity Index Map (SSIM), or Correlation Coefficient (CORR), with all values the larger, the better.

On the other hand, one may be interested in how “noisy” the image really is. The level of noise is commonly measured by Contrast to Noise Ratio (CNR—the larger the better), requiring the assignment of a region of interest and surrounding tissue (background). If the artifacts are present, one may calculate the intensity of the artifact signal, called the Artifact Index (AIx—the lower the better). Alternatively, one can calculate the Differentiation between Voxel Values (ΔVV—the lower the better)—the absolute difference in voxel values between a region with the most pronounced artifact and a corresponding artifact-free control region.

In deep learning model training, one needs a “ground truth” objects, to which one compares the result of the model. As the results are usually images, one can measure the difference in obtained and required intensity pixel-by-pixel, calculating either Mean Absolute Error (MAE), Mean Squared Error (MSE), Root Mean Square Error (RMSE), or finally Normalized Root Mean Square Error (NRMSE). In each case, lower values are better, as they denote closer similarity to the desired outcome of the model.

It has to be noticed that better values of some metrics, like PSNR, do not automatically mean better perceived visual quality, especially in images with structural or perceptual differences. Therefore, the independent assessment of an expert in the field is also highly desirable, especially in the denoising process, where the expected outcome is not known. In particular, in most studies at least two experts were involved (Table 1).

In what follows, there is a description of the methods used and results obtained in each group of articles. The key values of metrics in each work, along with the reference methods and image enhancement, are presented in Table 4.

### 3.5. Classical Methods in Image Denoising

A review of the literature reveals that, as of yet, there are only two articles directly treating the problem of CBCT image denoising; however, their objectives and methodology are different. The first article aims to help in dental prostetics by denoising the CBCT images with the introduced Sampling Kantorovich (SK) algorithm [27]. The SK algorithm is a very precise mathematical operation, working as a filter, reconstructing the image with the noise smoothed out, but without distortion of the main subject or adding artifacts. The CBCT images denoised with the SK algorithm were compared to other standard denoising (bilinear and bicubic B-spline), showing that, e.g., the edges of the dental arch, paramount in prostetics, were more visible.

The other article describes the Iterative Noise Reduction (INR) introduced using Fuzzy-Entropic-based algorithms [35]. The aim of the study was to assess the root canal therapy outcomes, where the denoised CBCT images were compared to Digital Periapical Film (DPF) measurements. As it turned out, the denoised CBCT images were superior to DPF, showing statistically significant differences in assessing root canal filling length, root canal closeness, and root canal filling quality.

The denoising was also quantified objectively by calculating PSNR (both articles) and CORR (Zhang et al. [35]) metrics. However, the definition of PSNR used in both methods differs by a constant factor 10·log(width·height) taking into account the image width and height, not mentioned in the source article [27]. Assuming both dimensions were of the size of $10^{3}$ pixels, the values of PSNR in Zhang et al. are shifted by a value around 90 dB. Yet, it seems that even after subtracting the correction, the PSNR values are higher for INR than for the SK algorithm (see Table 5). The efficiency of the algorithms was also compared to classical methods; however, the reference method differed between the papers. In particular, the SK algorithm has around 12% better PSNR than the B-spline method, while INR is around 3% better in terms of PSNR than the method based on the Penalized Weighted Least Squares algorithm (PWLS), which is only a part of the full INR method (Table 4).

### 3.6. Evaluation of AI-Based Denoising Models

In a series of three papers, the authors performed denoising of CBCT images using the vendor-agnostic model ClariCT.AI from ClariPi (Seoul, Republic of Korea) [28,29,33]. The ClariCT.AI model is founded on a Convolutional Neural Network (CNN) algorithm that reduces noise, and it incorporates Digital Imaging and Communications in Medicine (DICOM)-based sinogram blending and crossover inverse ray (IR) [37]. The authors conducted a comprehensive study, assessing the general image enhancement measured by CNR (evaluated on 3 regions of interest) [29], the image quality in CBCT images specifically focused on the temporomandibular joints (TMJs) [28], and the reduction in metal artifacts [33].

In each case, the utilization of ClariCT led to a reduction in noise, resulting in an enhancement of CNR (see Table 4). Yet, the subjective analysis was more nuanced. In particular, in both Kazimierczak’s et al. studies [28,29], although the AI-reconstructed images were usually preferred by the experts, there were no statistically significant differences in subjective image quality assessments. Conversely, Wajer’s study [33] demonstrated that subjective assessments exhibited a preference for DLM images, with higher ratings allocated to overall image quality, although, apart from CNR, other metrics did not show significant image enhancement. In particular, the ΔVV was similar after denoising, and the AIx values were better, but not close to the metal artifact itself, which was still intense. Yet, the image was cleaner and less distracting, with the halo around the artifact cleaned out. It seems that although the objective AI increases CNR and reduces noise, it does not automatically mean the image is better for diagnosis.

### 3.7. Transforming Images Between Techniques

The largest group contains articles introducing novel deep learning models, enhancing the image quality. The models, however, differ in main goal, architecture, solved task, training regime, and the loss function. The main details of each model are summarized in Table 5.

Regarding the aim, half of the models were thought to lower the exposure radiation. In particular, Zhao et al. [36] introduce a model called VVBPNet (View-by-view Backprojection Network), whose aim was to reconstruct the high-quality CBCT image from sparse data. On the other hand, Vestergaard et al. [32] introduce a model designed for the downstream task of improving the proton dose calculation, while the model of Ryu S. et al. [31] was introduced to apply automatic airway segmentation for Computational Fluid Dynamics (CFD) analysis. Only in one case was the task realized by the model just denoising the image [15]. In this case, the realistic noise was added to high-quality CBCT, and the model’s objective was to remove that noise and recreate CBCT images after the penalized least squares method with total variation regularization (PLS-TV).

Three other models aimed at creating (synthetic) CT images from CBCT scans [31,32,34]. In such a setup, there is always a problem to accurately match the images, as they are usually shifted due to another patient position and patient movement. This was solved by either aligning specific skull landmarks between the CT and CBCT scans [31] or by the deformable registration of the images [32,34]. The task of COMPUNet (Contextual Loss-Optimized Multi-Planar 2.5D U-Net) by Ryu K. et al. [30] was to recreate Multidetector Computed Tomography (MDCT) images from standard CBCT images measured at the same time, and VVBPNet by Zhao et al. [36] aimed at reconstruction of high-quality images from sparse data.

The model architecture also varied. In most cases it was a variation on a UNet architecture [15,30,31,34,36], or the model was trained in the Generative Adversarial Network (GAN) fashion [31,32,34]. UNet (named after the pictorial representation of the architecture in the U-shape) was introduced specifically for biomedical image segmentation tasks. It consists of encoder and decoder paths, which, working together, transform one image into another. In GAN models, one trains generator and discriminator models. The aim of the generator is to create a better image, while the quality of the image is judged by the discriminator. An interesting alternative is the CycleGAN network used by Vestergaard et al. [32], where the CBCT images are translated to CT and back to CBCT images, and the consistency between the original and recreated CBCT images is checked. Two other architectures proposed by Vestergaard et al. were the Contrastive Unpaired Translation (CUT) network, which maximizes the mutual information between corresponding patches or volumes in unpaired CBCT and CT images, and the mixture of the two (CycleCUT). More complex architecture was proposed by Zhao et al. [36] in the VVBPNet model, where the input data are the view-by-view backprojection tensors, which are intermediate results in the FDK algorithm. Those tensors are then applied to the network, which consists of two UNet-based paths, one for learning content and one for noise. Other architectures used are conditional GAN (cGAN), where, conversely to normal GAN, the discriminator also has access to the input CBCT image, and Unsupervised Image-to-Image Translation Network (UNIT), which works efficiently in the GAN manner.

In the training of deep learning models, what drives the model to the desired output is the definition of the loss function. In most cases it is based on Mean Absolute Error (MAE) between the intensity of the pixels in the Ground Truth and output images. In some cases, however, the loss was more complicated. In particular, Ryu K. et al. [30], apart from MAE, also tested VGG loss, which measures the similarity between internal representations of the images, and in the final model used the combination of both factors. A similar approach was also used by Ryu K. et al. [30]. On the other hand, the loss in the GAN network requires the effect of the generator and discriminator. To this end, Vestergaard et al. [32] used a deeply specialized loss based on cross-entropy.

Apart from the loss function, the size of the datasets and the diversity between images are important. The sizes of the training datasets are very small, only in one case exceeding 100 cases (Table 5). Additionally, in certain instances, the network’s performance was not evaluated using a held-out dataset. Consequently, despite the potentially favorable metrics, the actual validity of the networks and their potential to overcome common CBCT problems may be constrained. Surprisingly, only in two articles was the training dataset augmented by adding some noise [30,31], and in one case the model was previously pretrained on a larger set of images [30]. As none of the articles measured the similarity between the images, the existence of data leaks was not quantified. Moreover, it cannot be tested post factum, as there is no information if any model or dataset is placed in any public repository. Therefore, the models, along with their metrics, cannot be directly compared, as some problems solved may be easier due to less diverse test sets. This can also explain different results in subjective evaluation by experts. In some cases, the verdict of the experts was in favor of the Deep Learning Model [30,32], but in some cases the generated images were blurred and inferior to standard methods [15,34].

## 4. Discussion

### 4.1. Comparison of Traditional and AI-Based Tools

The main findings of the review are shown in Table 6. The present systematic review demonstrated that a statistically significant enhancement in CBCT image quality parameters was accomplished by the algorithms employed in each of the articles reviewed. However, direct comparison of the methods seems impossible due to the different reference methods and objectives. In particular, the widely used PSNR metric depends heavily on the input images. Nevertheless, it seems that the best results in image denoising were obtained by Ylisiurua et al., who reported the PSNR on the level of 70 dB, with a relative increase in over 50% compared to the standard algorithm. Still, one has to bear in mind that in this study there was no proper held-out test group in the model training. Yet another notable result is that of Costarelli et al. [27], who noted a PSNR of around 50 dB with the use of the Sampling Kantorovich method. This is higher than in most Deep Learning models, which obtain the PSNR value of around 30 dB. A comparison of these results with the current knowledge on CT examinations reveals a similarity in outcomes, with AI demonstrating a significant capacity to enhance image quality through denoising [6,38,39,40].

The literature on denoising methods in CBCT is limited, and a significant group of the articles we reviewed and excluded were phantom studies. Most of the proposed methods have not been widely implemented for clinical use. The selected set of articles describes three different areas where the CBCT denoising is analyzed. It is either in the case of classical methods, where the denoising algorithm is rather known and what can be enhanced is the fidelity of the transformed figure. The other group is analysis of the potency of existing Deep Learning models in denoising or removing artifacts. The last group is the creation of new models capable of transforming CBCT images to different techniques (usually CT). Conventional analytical reconstruction algorithms, such as FBP, are still mainly used in dental practice [41], which demonstrates their high utility but also the need for further research into AI denoising tools. IR algorithms, despite their good performance in quality enhancement parameters, often prove to be diagnostically impractical, as 3D raw CBCT data impose increased memory requirements and impractical computation times in a clinical setting [42,43]. In addition, literature data from CT studies showed that MBIR models reduce noise more than FBP [44,45,46], but the image extracted from these reconstructions is described as plastic and artificial [46] and further hinders the detection of small tissue differences, especially at low contrast [47].

Also, Deep Learning models have been shown to improve overall image quality significantly and better than MBIR or FBP in many low-dose CT studies [48,49,50]. From this overview, it can be concluded that Deep Learning models in CBCT provide the same or similar or even better noise reduction and overall image quality than FBP or IR. Since Deep Learning-based reconstruction or noise reduction is not an iterative process, the computation time can be reduced compared to IR, which might be the most important improvement.

Despite their benefits, literature on AI denoising techniques faces several limitations. One of the main challenges is the lack of standardized parameters for assessing image quality. Different studies use different metrics, making it difficult to make comprehensive comparisons and determine the most effective method. Many studies have been conducted on small cohorts of patients, which limits the generalizability of the results. Moreover, in some cases the experts judged the AI-denoised image as inferior compared to standard methods in some aspects. Therefore, although the quantitative metrics may be promising, the Deep Learning denoising might lose some important features of the analyzed images, which might for now be ineffective in an everyday practice.

The effectiveness of AI models largely depends on the availability of high-quality training data, which is not always available. Dose reduction in CBCT, like CT, can produce images of very poor quality, which can lead to DLR artifacts called hallucinations—when the network incorrectly identifies noise as a missing object and replaces it with a non-existent structure— or inverted hallucinations—when the network incorrectly removes a section [51,52]. Although such cases were not directly reported in the analyzed study, there were some Deep Learning-specific problems mentioned. For example, Ryu K. et al. [30] mention erroneous background masking and streaking artifacts. Therefore, it is important to explore this in more depth before DLR is widely used in clinical practice. Moreover, it is worth noting that in some cases the results differ between anatomical regions. For example, the morphology of the nasal cavity was found to be challenging to capture accurately, and some blurring effects were spotted in the hypopharynx and oropharyngeal regions [31]. On the other hand, in some cases the model enhanced the visibility, especially of the sinus floor and the TMJ complex [30]. Both effects can be, however, the results of the training set, where those regions could be represented with different accuracy.

With the increased availability of photo-counting CT scanners, the data obtained will be of high quality, which is likely to significantly improve the quality of the images, and studies relating to the use of PCCT’s in dentistry can be expected [53,54]. Sawal et al. [55], in their study, showed that, compared to conventional dental digital volumetric tomography systems, photon-counting computed tomography provides higher image quality even at lower dose levels. In this study, CNRD is higher in all PCCT acquisitions, and CNRD (normalized contrast-to-noise ratio) is 37% higher for dentin-enamel contrast and 31% higher for dentin-bone contrast.

### 4.2. Limitations

A number of limitations to this systematic review must be acknowledged. Firstly, the selection criteria employed a stringent approach, including exclusively articles published in peer-reviewed journals and indexed in prominent databases, written in English. This approach may have introduced publication bias. Furthermore, given that the application of artificial intelligence to CBCT exams is typically above standard but also technically unchallenging, the possibility of publication bias is heightened. Consequently, there is a possibility that some single-center experiences have not been published. While the peer-review system does not ensure optimal scientific value, its formal requirements should enhance the quality of publications. Secondly, the present study encompasses a broad spectrum of software postprocessing methodologies. Consequently, the accurate classification of noise reduction may be subject to bias. Finally, the absence of a uniform definition of significant noise reduction among the studies underscores the current paucity of high-quality evidence in this domain. Moreover, in some cases the definitions of the metrics used differed, and in some cases the definitions were not given. Another major obstacle is the lack of data or models publicly available. In some cases, the details of the model architecture remain unknown, and because of a lack of data, the results cannot be reobtained independently. Moreover, no similarity between images was measured; therefore, the capabilities of the Deep Learning models tested are practically incomparable.

### 4.3. Perspectives

In the current review, it turns out that most techniques used are based on some Deep Learning models. Analyzing the current trends and results, it seems that there is still room for improvement in this area. In particular, one might use some novel transformer-based techniques, like Denoising Vision Transformer. Another approach would be to use some foundational model pretrained on a large set of images either to obtain the correct internal representation or specifically for the denoising task. Moreover, due to the sparse set of images, it seems imperative to apply some dataset augmentation by adding artificial noise, random movements, rotations, etc. It would also be good to create a centralized dataset of the CBCT images, which could be used for training some models. In the best-case scenario, the images should be split into train/test/valid sets, based on reasonable criteria following from the image similarity. Such an approach would allow for a fair comparison of models.

The comparison should be performed with some standardized set of techniques. The absolute minimum in the area of image denoising is a metric showing the level of noise and the quantification of the similarity of important features with the reference figure. The latter can be measured using the SSIM metric. The former is usually measured with PSNR; however, this metric is also dependent on the reference images, so it does not calculate the noise level directly. It seems that metrics like Contrast-to-Noise Ratio (CNR) would better quantify the noise level. Also, to avoid any misunderstandings and provide a fair comparison, the metrics should be calculated according to the same equation included in the article. In the case of new Deep Learning models, it would be good if their code was publicly available, preferably with the datasets used. This would remove all the ambiguities related to the model’s architecture.

## 5. Conclusions

The development and clinical application of IR and DLM noise reduction algorithms have enabled patients to undergo examination at significantly reduced radiation dose levels. Due to their capacity to mitigate noise and produce diagnostic-quality CBCT images at reduced doses, IR and DL algorithms are anticipated to become the prevailing technique for reconstructing CBCT images at lower doses, displacing conventional FBP. In the pursuit of CBCT examinations at reduced doses, it is imperative that radiologists and dentists possess a comprehensive understanding of the strengths and limitations of IR and noise reduction algorithms. Moreover, they should be well-versed in the methodologies employed for the rigorous evaluation of these techniques.

## Figures and Tables

**Table 1:** Characteristics of the included studies.

	Study	Year	Country	Study Type/ No. of Patients	Reference Standard—Quantitative Evaluation	Reference Standard—Qualitative Evaluation	Anatomical Region
1.	Costarelli et al. [27]	2021	Italy	Patients/2	MSE, PSNR	NA	dental-maxillofacial region
2.	Kazimierczak et al. [28]	2024	Poland	Patients/50	CNR	A radiologist and orthodontist	temporomandibular joints
3.	Kazimierczak et al. [29]	2024	Poland	Patients/93	CNR	A radiologist and two dentists	dental-maxillofacial region
4.	Ryu K. et al. [30]	2023	South Korea, USA	Patients/30 Phantom/6	MAE, NRMSE, SSIM	Two radiologists	head and neck
5.	Ryu S. et al. [31]	2025	South Korea, USA	Patients/33	MAE, PSNR, SSIM	Unspecified researchers	head and neck
6.	Vestergaard et al. [32]	2024	Denmark	Patients/102	PSNR, SSIM, MAE, ME	NA	head and neck
7.	Wajer et al. [33]	2024	Poland	Patients/61	CNR, ΔVV, AIx	A radiologist and a dentist	dental-maxillofacial region
8.	Ylisiurua et al. [15]	2024	Finland	Patients/32	SSIM, PSNR	One dentomaxillofacial radiologist	dental-maxillofacial region
9.	Zhang Y. et al. [34]	2022	China	Patients/120	MAE, RMSE, SSIM, PSNR	NA	head and neck
10.	Zhang K. et al. [35]	2022	China	Patients/88	PSNR, CORR	NA	affected teeth
11.	Zhao et al. [36]	2025	China	Patients/223	RMSE, PSNR, SSIM, FSIM	NA	head

(AIx—artifact index, CNR—contrast to noise ratio, CORR—correlation coefficient, FSIM—Feature Similarity Index, MAE—mean absolute error, NA—not assessed, NRMSE—normalized root-mean-square deviation, PSNR—peak signal-to-noise ratio, RMSE—root mean square error, SSIM—Structural Similarity Index, ΔVV—differentiation between voxel values).

**Table 2:** Characteristics of a separate group of the included studies.

Study	Year	Anatomical Region	Model Used
Group 1—Denoising by classical methods (iterative reconstructions, filtering algorithms)			
Costarelli et al. [27]	2021	dental-maxillofacial region	Sampling Kantorovich (SK)
Zhang K. et al. [35]	2022	affected teeth	INR algorithm-based CBCT
Group 2—Evaluation of AI-based denoising model			
Kazimierczak et al. [28]	2024	temporomandibular joints	ClariCT.AI (commercial)
Kazimierczak et al. [29]	2024	dental-maxillofacial region	ClariCT.AI (commercial)
Wajer et al. [33]	2024	dental-maxillofacial region	ClariCT.AI (commercial)
Group 3—Transforming images between techniques			
Ryu S. et al. [31]	2025	head and neck	CycleGAN
Vestergaard et al. [32]	2024	head and neck	CycleGAN/CUT
Zhang Y. et al. [34]	2022	head and neck	GAN
Ylisiurua et al. [15]	2024	dental-maxillofacial region	UNIT and U-Net
Ryu K. et al. [30]	2023	head and neck	UNet
Zhao et al. [36]	2025	head	VVBPNet

**Table 3:** Risk of bias assessment according to the QUADAS-2 tool. The high and unclear values are shown in bold.

Study	Risk of Bias				Applicability Concerns		
	Patient Selection	Index Test	Reference Standard	Flow and Timing	Patient Selection	Index Test	Reference Standard
Costarelli et al., 2021 [27]	**High**	**Unclear**	Low	**High**	**High**	Low	Low
Kazimierczak et al., 2024 [28]	Low	Low	Low	Low	Low	Low	Low
Kazimierczak et al., 2024 [29]	Low	Low	Low	Low	Low	Low	Low
Ryu K. et al., 2023 [30]	Low	Low	Low	Low	Low	Low	Low
Ryu S. et al., 2025 [31]	Low	Low	Low	Low	Low	Low	Low
Vestergaard et al., 2024, [32]	Low	Low	Low	Low	Low	Low	Low
Wajer et al., 2024 [33]	Low	Low	Low	Low	Low	Low	Low
Ylisiurua et al., 2024 [15]	Low	Low	Low	Low	Low	Low	Low
Zhang Y. et al., 2022 [34]	Low	Low	Low	Low	Low	Low	Low
Zhang K. et al., 2022 [35]	Low	Low	Low	Low	Low	Low	Low
Zhao et al., 2025 [36]	**Unclear**	**Unclear**	Low	Low	**Unclear**	Low	Low

**Table 4:** Comparative parameters of denoising models. The values were divided into the ones that are calculated in comparison with Ground Truth images and with results of a reference, classical method if present. For some metrics (PSNR, CORR), the metric is calculated in reference to Ground Truth. For others (CNR), the value of the metric can be calculated for Ground Truth and for results separately, allowing for comparison of values. The means are calculated for each Region of Interests specified in the source article, without reweighting due to different numbers of scans. Rel. metric enhancement is the metric enhancement in reference to the ground truth or reference method. Only the metrics concerning image enhancement are considered. * in Zhang K. et al. [35], the PSNR metric was calculated differently than in other works, as a result being shifted by around 90 dB. ** in Wajer et al. [33], the CNR metric was calculated differently, with noise of artifact and control separated.

Study	Algorithm Name	Comparison with Ground Truth				Comparison with Classical Method	
		Ground Truth Images	Metric	Mean Metric Value	Rel. Metric Enhancement	Reference Method	Rel. Metric Enhancement
Classical image denoising							
Costarelli et al. [27]	Sampling Kantorovich (SK)	Original CBCT images	PSNR	58.1 dB	---	Bilinear B-spline	15.5%
						Bicubic B-spline	13.8%
Zhang K. et al. [35]	Iterative Noise Reduction (INR)	Original CBCT images	PSNR (shifted)	191 dB	---	PWLS	2.1%
			PSNR—90 dB *	101 dB	---		4.1%
			CORR	0.993	---		0.3%
Evaluation of AI-based denoising							
Kazimierczak et al. [28]	ClariCT.AI	Original CBCT images	CNR	11.03	44.8%	---	---
Kazimierczak et al. [29]	ClariCT.AI	Original CBCT images	CNR	9.92	35.6%	---	---
Wajer et al. [33]	ClariCT.AI	Original CBCT images	CNR **	0.93	17.2%	---	---
			AIx	350.92	−5.0%		
			ΔVV	341.04	−0.2%		
Transforming images between techniques							
Ylisiurua et al. [15]	UNet	CBCT scans after PLS-TV regularization	PSNR	77.4 dB	---	FDK denoised images	52.9%
			SSIM	1.0	---		7.5%
	UNIT		PSNR	74.6 dB	---		47.4%
			SSIM	1.0	---		7.5%
Ryu K. et al. [30]	COMPUNet	MDCT images	NRMSE	0.14	---	Comparison with original CBCT	35.7%
			SSIM	0.84	---		10.5%
Ryu S. et al. [31]	CycleGAN with MAEVGG loss	Ground truth CT scans	PSNR	28.65 dB	---	Comparison with original CBCT	28.3%
			SSIM	0.87	---		40.2
Vestergaard et al. [32]	CycleGAN	Ground truth CT scans	PSNR	31.8 dB	---	Comparison with original CBCT	24.2%
			SSIM	0.97	---		2.1%
	CUT		PSNR	31.8 dB	---		24.2%
			SSIM	0.97	---		2.1%
	CycleCUT		PSNR	31.8 dB	---		24.2%
			SSIM	0.97	---		2.1%
Zhang Y. et al. [34]	cGAN	Reference CT images	PSNR	30.58 dB	---	Comparison with original CBCT	20.7%
			SSIM	0.90	---		8.4%
	CycleGAN		PSNR	29.29 dB	---		15.6%
			SSIM	0.92	---		10.8%
	UNet		PSNR	30.48 dB	---		20.3%
			SSIM	0.90	---		8.4%
Zhao et al. [36]	VVBPNet	Reconstructed from full view projections	PSNR	37.3 dB	---	FDK denoised images	21.9%
			SSIM	0.90	---		26.4%
			FSIM	0.99	---		0.1%

**Table 5:** Deep learning characteristics of studies describing image transformation methods.

Study	Task	Model Architectures	Dataset Size (Train/Valid/Test)
Ryu K. et al. [30]	CBCT -> MDCT	UNet	30 (30/0/0)
Ryu S. et al. [31]	CBCT -> CT	CycleGAN	33 (22/0/11)—cross validation
Vestergaard et al. [32]	CBCT -> CT	CycleGAN, CUT, CycleCut	102 (77/5/20)
Zhang et al. [34]	CBCT -> CT	GAN	120 (80/10/30)
Ylisiurua et al. [15]	Simulated CBCT -> CBCT	UNet, UNIT	22 (22/0/0)
Zhao et al. [36]	Sparse CBCT -> CBCT	UNet	223 (163/30/30)

**Table 6:** Main finding of the review.

Category	Classic Method	Deep Learning Model
Quantitative analysis	New algorithms (SK, INR) perform much better than older ones.	Models performing resonably good in denoising images.
Subjective anaysis	The images cleaner, however, the sample of methods is small.	Mixed feelings—in most cases the images are smoother, clearner, brighter, yet in some cases the experts prefered the output of classic methods.
Time of analysis	Rather slow.	Speed up 1–2 orders of magnitude.
Usage	Denoising, further downstream tasks.	Obtaining synthetic images from different techniques, more precise radiation dose calculation, lowering dose using sparse view.

## Data Availability

Data are available upon request.

## Supplementary Materials

The following supporting information can be downloaded at: https://www.mdpi.com/article/10.3390/jcm14124214/s1, Table S1: Studies excluded after full-text analysis [41,56,57,58,59,60,61,62,63,64,65,66,67,68,69,70,71,72,73,74,75,76,77,78,79]. Table S2: Exact phrases used in the search engines.

## Abbreviations

CNN = convolutional neural network, CBCT = cone beam computed tomography, DLR = deep learning reconstruction, FBP = filtered back projection, FDA = U.S. Food and Drug Administration HIR = hybrid iterative reconstruction, IR = iterative reconstruction, MAR = metal artifact reduction, AI = artificial intelligence.
