# Supplementary material for: Enhancing Image Quality in Dental-Maxillofacial CBCT: The Impact of Iterative Reconstruction and AI on Noise Reduction—A Systematic Review
Source: J Clin Med. 2025 Jun 13;14(12):4214. doi: 10.3390/jcm14124214 (PMC12194620; doi:10.3390/jcm14124214)
Supplement: Supplementary file 1 [file jcm-14-04214-s001.zip › jcm-3631468-supplementary.pdf]

Table S1. Studies excluded after full-text analysis

No.	Title	Year	Journal	Authors	Search engine	Language	Reason for exclusion
1.	Towards dental diagnostic systems: Synergizing wavelet transform with generative adversarial networks for enhanced image data fusion [1]	2024	Computers in Biology and Medicine	Al-Haddad, Abdullah A.,	ScienceDirect	English	studies not assessing diagnostic dental or maxillofacial CBCT
2.	Development of a New Cone-Beam Computed Tomography Software for Endodontic Diagnosis [2]	2018	Brazilian dental journal	Bueno, M. R.	Pubmed	English	non-ORIGINAL and research studies,
3.	Root Canal Shape of Human Permanent Teeth Determined by New Cone-Beam Computed Tomographic Software [3]	2020	Journal of Endodontics	Bueno, Mike R.	ScienceDirect	English	studies not assessing diagnostic dental or maxillofacial CBCT
4.	Generative Noise Reduction in Dental Cone-Beam CT by a Selective Anatomy Analytic Iteration Reconstruction Algorithm [4]	2019	ELECTRONICS	Dao-Ngoc, L.		English	studies not involving human subjects
5.	Enhancement of three-dimensional medical images [5]	2024	Advances in Computers	Dhananjay, B.		English	Book chapter
6.	Iterative tomographic reconstruction with TV prior for low-dose CBCT dental imaging [6]	2022	Phys Med Biol	Friot-Giroux, L.	Pubmed	English	studies not involving human subjects
7.	Concrete learning method for segmentation and denoising using CBCT Image [7]	2023	ACM International Conference Proceeding Series	Gan, J.		English	Conference Paper
8.	Half-scan artifact correction using generative adversarial network for dental CT [8]	2021	Computers in Biology and Medicine	Hegazy, Mohamed A. A.	Pubmed	English	studies not involving human subjects
9.	StarAN: A star attention network utilizing inter-view and intra-view correlations for	2024	Expert Systems with Applications	Jin, Xin	ScienceDirect	English	studies not involving human subjects

	sparse-view cone-beam computed tomography reconstruction [9]						
10.	Enhanced artificial intelligence-based diagnosis using CBCT with internal denoising: Clinical validation for discrimination of fungal ball, sinusitis, and normal cases in the maxillary sinus [10]	2023	Computer Methods and Programs in Biomedicine	Kim, Kyungsu	ScienceDirect	English	studies not assessing diagnostic dental or maxillofacial CBCT
11.	Optimized Anisotropic Rotational Invariant Diffusion Scheme on Cone-Beam CT [11]	2010	MEDICAL IMAGE COMPUTING AND COMPUTER-ASSISTED INTERVENTION - MICCAI 2010, PT III	Kroon, D. J.	ScienceDirect	English	Conference Paper
12.	Automatic tooth arrangement with joint features of point and mesh representations via diffusion probabilistic models [12]	2024	Computer Aided Geometric Design	Lei, Changsong	ScienceDirect	English	studies not assessing diagnostic dental or maxillofacial CBCT
13.	Eliminating metal artifacts in dental computed tomography using an elaborate sinogram normalization interpolation method with CNR-based metal segmentation [13]	2024	Journal of Instrumentation	Lim, Y.		English	studies not involving human subjects
14.	An Efficient Quality Enhancement Method for Low-Dose CBCT Imaging [14]	2025	WSEAS Transactions on Biology and Biomedicine	Mirzaei, S.		English	lacking information Ethical Board approval
15.	Development and Evaluation of a Deep Learning Model to Reduce Exomass-Related Metal Artifacts in Cone-Beam Computed Tomography of the Jaws [15]	2024	Dentomaxillofac Radiol	Oliveira, M. L.		English	studies not involving human subjects
16.	Unpaired-Paired Learning for Shading Correction in Cone-Beam Computed Tomography [16]	2022	IEEE ACCESS	Park, H. S.		English	studies not assessing diagnostic dental

							or maxillofacial CBCT
17.	Filtered back projection vs. iterative reconstruction for CBCT: effects on image noise and processing time [17]	2023	Dentomaxillofacial Radiology	Ramage, A.		English	studies not involving human subjects
18.	Comparison of two- and three-dimensional filtering methods to improve image quality in multiplanar reconstruction of cone-beam computed tomography [18]	2009	Oral Radiology	Sagawa, M.		English	lacking information Ethical Board approval
19.	CDRMamba: A framework for automated craniomaxillofacial defect reconstruction using Mamba-based modeling [19]	2025	Biomedical Signal Processing and Control	Wang, Wensheng	ScienceDirect	English	studies not assessing diagnostic dental or maxillofacial CBCT
20.	Variable-resolution cone-beam computerized tomography with enhancement filtration compared with intraoral photostimulable phosphor radiography in detection of transverse root fractures in an in vitro model [20]	2009	Oral Surgery, Oral Medicine, Oral Pathology, Oral Radiology, and Endodontology	Wenzel, Ann	ScienceDirect	English	studies not assessing diagnostic dental or maxillofacial CBCT
21.	Ultralow Dose MSCT Imaging in Dental Implantology [21]	2018	The Open Dentistry Journal	Widmann, Gerlig		English	review
22.	Clinical application of mixed reality holographic imaging technology in scaling and root planing of severe periodontitis: a proof of concept [22]	2024	Journal of Dentistry	Xue, Fei	ScienceDirect	English	studies not assessing diagnostic dental or maxillofacial CBCT
23.	Penalty-driven enhanced self-supervised learning (Noise2Void) for CBCT denoising [23]	2023	Progress in Biomedical Optics and Imaging - Proceedings of SPIE	Yun, S.		English	poster
24.	Scatter correction based on adaptive photon path-based Monte Carlo simulation method in Multi-GPU platform [24]	2020	Computer Methods and Programs in Biomedicine	Zhang, Yangmei	ScienceDirect	English	studies not assessing diagnostic dental

							or maxillofacial CBCT
25.	Texture-preserving diffusion model for CBCT-to-CT synthesis [25]	2025	Medical Image Analysis	Zhang, Youjian	ScienceDirect	English	studies not assessing diagnostic dental or maxillofacial CBCT

Table S2. Exact phrases used in the search engines

Search engine	Phrase
PubMed	("CBCT" OR "cone-beam computed tomography") AND ("denoising" OR "denoise" OR "noise reduction") AND (“oral cavity” OR “maxillofacial” OR “dental”)
Scopus	(TITLE-ABS-KEY(CBCT) OR TITLE-ABS-KEY("cone-beam computed tomography")) AND (TITLE-ABS-KEY(denoising) OR TITLE-ABS-KEY(denoise) OR TITLE-ABS-KEY(“noise reduction")) AND (TITLE-ABS-KEY(“oral cavity”) OR TITLE-ABS-KEY(“maxillofacial”) OR TITLE-ABS-KEY(“dental”))
Web of Science	TS=("CBCT" OR "cone-beam computed tomography") AND TS= ("denoising" OR "denoise" OR "noise reduction") AND TS=(“oral cavity” OR “maxillofacial” OR “dental”)
Embase	("cbct" OR "cone-beam computed tomography") AND ("denoising" OR "denoise" OR "noise reduction") AND (“oral cavity” OR “maxillofacial” OR “dental”)
Science Direct	("CBCT" OR "cone-beam computed tomography") AND ("denoising" OR "denoise" OR "noise reduction") AND (“oral cavity” OR “maxillofacial” OR “dental”)

References:

1. Al-Haddad, A.A.; Al-Haddad, L.A.; Al-Haddad, S.A.; Jaber, A.A.; Khan, Z.H.; Rehman, H.Z.U. Towards Dental Diagnostic Systems: Synergizing Wavelet Transform with Generative Adversarial Networks for Enhanced Image Data Fusion. *Comput Biol Med* 2024, *182*, 109241, doi:https://doi.org/10.1016/j.compbiomed.2024.109241.

2. Bueno, M.R.; Estrela, C.; Azevedo, B.C.; Diogenes, A. Development of a New Cone - Beam Computed Tomography Software for Endodontic Diagnosis. *Braz Dent J* 2018, *29*, 517–529, doi:10.1590/0103-6440201802455.

3. Bueno, M.R.; Estrela, C.; Azevedo, B.C.; Cintra Junqueira, J.L. Root Canal Shape of Human Permanent Teeth Determined by New Cone-Beam Computed Tomographic Software. *J Endod* 2020, *46*, 1662–1674, doi:https://doi.org/10.1016/j.joen.2020.05.014.

4. Dao-Ngoc, L.; Du, Y.-C. Generative Noise Reduction in Dental Cone-Beam CT by a Selective Anatomy Analytic Iteration Reconstruction Algorithm. *Electronics (Basel)* 2019, *8*, doi:10.3390/electronics8121381.

5. Budaraju, D.; C K, N.; Hiremath, B.; Ravi, P.; Lakshminarayana, M.; Neelapu, B.; Jayaraman, S. Enhancement of Three-Dimensional Medical Images. In; 2024 ISBN 9780323988575.

6. Friot--Giroux, L.; Peyrin, F.; Maxim, V. Iterative Tomographic Reconstruction with TV Prior for Low-Dose CBCT Dental Imaging. *Phys Med Biol* 2022, *67*, doi:10.1088/1361-6560/ac950c.

7. GAN, J.; Yu, N.; Qian, G.; He, N. Concrete Learning Method for Segmentation and Denoising Using CBCT Image. In Proceedings of the Proceedings of the 2023 4th International Conference on Control, Robotics and Intelligent System; Association for Computing Machinery: New York, NY, USA, 2023; pp. 41–46.

8. Hegazy, M.; Cho, M.; Lee, S. Half-Scan Artifact Correction Using Generative Adversarial Network for Dental CT. *Comput Methods Programs Biomed* 2021, *132*, 104313, doi:10.1016/j.compbiomed.2021.104313.

9. Jin, X.; Zhu, Y.; Wu, K.; Hu, D.; Gao, X. StarAN: A Star Attention Network Utilizing Inter-View and Intra-View Correlations for Sparse-View Cone-Beam Computed Tomography Reconstruction. *Expert Syst Appl* 2024, *258*, 125099, doi:10.1016/j.eswa.2024.125099.

10. Kim, K.; Lim, C.; Shin, J.; Chung, M.; Jung, Y.G. Enhanced Artificial Intelligence-Based Diagnosis Using CBCT with Internal Denoising: Clinical Validation for Discrimination of Fungal Ball, Sinusitis, and Normal Cases in the Maxillary Sinus. *Comput Methods Programs Biomed* 2023, *240*, 107708, doi:10.1016/j.cmpb.2023.107708.

11. Kroon, D.-J.; Slump, C.; Maal, T. *Optimized Anisotropic Rotational Invariant Diffusion Scheme on Cone-Beam CT*; 2010; Vol. 13; ISBN 978-3-642-15710-3.

12. Lei, C.; Mengfei, X.; Wang, S.; Liang, Y.; Yi, R.; Wen, Y.-H.; Liu, Y.-J. Automatic Tooth Arrangement with Joint Features of Point and Mesh Representations via Diffusion Probabilistic Models. *Comput Aided Geom Des* 2024, *111*, 102293, doi:10.1016/j.cagd.2024.102293.

13. Lim, Y.; Park, S.; Jeon, D.; Kim, W.; Lee, S.; Cho, H. Eliminating Metal Artifacts in Dental Computed Tomography Using an Elaborate Sinogram Normalization Interpolation Method with CNR-Based Metal Segmentation. *Journal of Instrumentation* 2024, *19*, C11003, doi:10.1088/1748-0221/19/11/C11003.

14. Mirzaei, S.; Tohidypour, H.R.; Nasiopoulos, P.; Mirabbasi, S. An Efficient Quality Enhancement Method for Low-Dose CBCT Imaging. *WSEAS TRANSACTIONS ON BIOLOGY AND BIOMEDICINE* 2024, *22*, 76–81, doi:10.37394/23208.2025.22.9.

15. Oliveira, M.; Schaub, S.; Dagassan-Berndt, D.; Bieder, F.; Cattin, P.; Bornstein, M. Development and Evaluation of a Deep Learning Model to Reduce Exomass-Related Metal Artefacts in Cone-Beam Computed Tomography of the Jaws. *Dentomaxillofac Radiol* 2024, *54*, doi:10.1093/dmfr/twae062.

16. Park, H.; Jeon, K.; Lee, S.-H.; Seo, J. Unpaired-Paired Learning for Shading Correction in Cone-Beam Computed Tomography. *IEEE Access* 2022, *10*, 26140–26148, doi:10.1109/ACCESS.2022.3155203.

17. Ramage, A.; Gutierrez, B.; Fischer, K.; Sekula, M.; Santaella, G.; Scarfe, W.; Brasil, D.; Oliveira-Santos, C. Filtered Back Projection vs. Iterative Reconstruction for CBCT: Effects on Image Noise and Processing Time. *Dentomaxillofac Radiol* 2023, *52*, 20230109, doi:10.1259/dmfr.20230109.

18. Sagawa, M.; Miyoseta, Y.; Hayakawa, Y.; Honda, A. Comparison of Two - and Three-Dimensional Filtering Methods to Improve Image Quality in Multiplanar Reconstruction of Cone-Beam Computed Tomography. *Oral Radiol* 2009, *25*, 154–158, doi:10.1007/s11282-009-0026-9.

19. Wang, W.; Jin, Z.; Chen, X. CDRMamba: A Framework for Automated Craniomaxillofacial Defect Reconstruction Using Mamba-Based Modeling. *Biomed Signal Process Control* 2025, *103*, 107376, doi:https://doi.org/10.1016/j.bspc.2024.107376.

20. Wenzel, A.; Haiter-Neto, F.; Frydenberg, M.; Kirkevang, L.-L. Variable-Resolution Cone-Beam Computerized Tomography with Enhancement Filtration Compared with Intraoral Photostimulable Phosphor Radiography in Detection of Transverse Root Fractures in an in Vitro Model. *Oral Surgery, Oral Medicine, Oral Pathology, Oral Radiology, and Endodontology* 2009, *108*, 939–945, doi:https://doi.org/10.1016/j.tripleo.2009.07.041.

21. Widmann, G.; Al-Ekrish, A.A. Ultralow Dose MSCT Imaging in Dental Implantology. *Open Dent J* 2018, *12*, 87–93, doi:10.2174/1874210601812010087.

22. Xue, F.; Zhang, R.; Dai, J.; Zhang, Y.; Luan, Q.-X. Clinical Application of Mixed Reality Holographic Imaging Technology in Scaling and Root Planing of Severe Periodontitis: A Proof of Concept. *J Dent* 2024, *149*, 105284, doi:https://doi.org/10.1016/j.jdent.2024.105284.

23. Yun, S.; Jeong, U.; Kwon, T.; Choi, D.; Lee, T.; Ye, S.-J.; Cho, G.; Cho, S. Penalty-Driven Enhanced Self-Supervised Learning (Noise2Void) for CBCT Denoising. In Proceedings of the Proc.SPIE; April 7 2023; Vol. 12463, p. 1246327.

24. Zhang, Y.; Chen, Y.; Zhong, A.; Jia, X.; Wu, S.; Qi, H.; Zhou, L.; Xu, Y. Scatter Correction Based on Adaptive Photon Path-Based Monte Carlo Simulation Method in Multi-GPU Platform. *Comput Methods Programs Biomed* 2020, *194*, 105487, doi:https://doi.org/10.1016/j.cmpb.2020.105487.

25. Zhang, Y.; Li, L.; Wang, J.; Yang, X.; Zhou, H.; He, J.; Xie, Y.; Jiang, Y.; Sun, W.; Zhang, X.; et al. Texture-Preserving Diffusion Model for CBCT-to-CT Synthesis. *Med Image Anal* 2025, *99*, 103362, doi:https://doi.org/10.1016/j.media.2024.103362.
